# The extracellular proteome of two *Bifidobacterium* species reveals different adaptation strategies to low iron conditions

**DOI:** 10.1186/s12864-016-3472-x

**Published:** 2017-01-06

**Authors:** Pamela Vazquez-Gutierrez, Marc J. A. Stevens, Peter Gehrig, Simon Barkow-Oesterreicher, Christophe Lacroix, Christophe Chassard

**Affiliations:** 1 Laboratory of Food Biotechnology, ETH Zurich, Institute of Food, Nutrition and Health, Schmelzbergstrasse 7, 8092 Zurich, Switzerland; 2 Functional Genomics Center Zurich, Winterthurerstrasse 190, 8057 Zurich, Switzerland; 3 Present Address: Institut National de la Recherche Agronomique, UR 545 URF, 15000 Aurillac, France

**Keywords:** *Bifidobacterium kashiwanohense*, *Bifidobacterium pseudolongum*, Iron binding, Genomics, Proteomics

## Abstract

**Background:**

Bifidobacteria are among the first anaerobic bacteria colonizing the gut. Bifidobacteria require iron for growth and their iron-sequestration mechanisms are important for their fitness and possibly inhibit enteropathogens. Here we used combined genomic and proteomic analyses to characterize adaptations to low iron conditions of *B. kashiwanohense* PV20-2 and *B. pseudolongum* PV8-2, 2 strains isolated from the feces of iron-deficient African infants and selected for their high iron-sequestering ability.

**Results:**

Analyses of the genome contents revealed evolutionary adaptation to low iron conditions. A ferric and a ferrous iron operon encoding binding proteins and transporters were found in both strains. Remarkably, the ferric iron operon of *B. pseudolongum* PV8-2 is not found in other *B. pseudolongum* strains and likely acquired via horizontal gene transfer. The genome *B. kashiwanohense* PV20-2 harbors a unique region encoding genes putatively involved in siderophore production.

Additionally, the secretomes of the two strains grown under low-iron conditions were analyzed using a combined genomic-proteomic approach. A ferric iron transporter was found in the secretome of *B. pseudolongum* PV8-2, while ferrous binding proteins were detected in the secretome of *B. kashiwanohense* PV20-2, suggesting different strategies to take up iron in the strains. In addition, proteins such as elongation factors, a glyceraldehyde-3-phosphate dehydrogenase, and the stress proteins GroEL and DnaK were identified in both secretomes. These proteins have been previously associated with adhesion of lactobacilli to epithelial cells.

**Conclusion:**

Analyses of the genome and secretome of *B. kashiwanohense* PV20-2 and *B. pseudolongum* PV8-2 revealed different adaptations to low iron conditions and identified extracellular proteins for iron transport. The identified extracellular proteins might be involved in competition for iron in the gastrointestinal tract.

**Electronic supplementary material:**

The online version of this article (doi:10.1186/s12864-016-3472-x) contains supplementary material, which is available to authorized users.

## Background

Iron is an essential micronutrient for most organisms and is taken up by high affinity transport systems [1]. Two oxidized forms of iron, ferric (Fe^3+^) and ferrous iron (Fe^2+^), occur in nature with ferrous iron being to be most abundant in the intestine [2]. Iron is limited in most environments and a battle for iron occurs in many microbial ecosystems, including the human gastro-intestinal (GI) tract [3, 4]. Withholding iron is therefore a competitive and defense trait in many Gram-positive and Gram-negative bacteria [5, 6]. Predominant members of an ecosystem frequently possess efficient iron-scavenging systems which enable them to outcompete other microorganisms by depriving them from iron [7]. In addition, restricting iron to pathogens in the GI-tract has been coined as nutritional immunity phenomenon and is usually associated with efficient iron-sequestration systems.

Bifidobacteria are non-sporeforming, nonmotile, anaerobic, Gram-positive bacteria with a high G + C content that are residents of the GI-tract and predominant in infants [8]. Bifidobacteria have been shown to beneficially modulate the composition and activity of the intestinal microbiota, to prevent bacterial infections, and to exert anti-inflammatory and immunomodulation activities [9]. These beneficial traits depend strongly on the ability of a strain to survive and to adapt in the GI-tract [10]. Adaptation and survival involve the use of efficient and diverse nutrient uptake systems, enzymes, stress proteins, and factors that interact with the host and with other members of the gut microbiota [11].

Bifidobacteria require iron for growth and produce extracellular binding proteins that are involved in iron uptake [12]. Such iron-binding proteins may also be implicated in iron withholding thereby limiting the availability to pathogens in the GI-tract [13]. Additionally, the essential micronutrient zinc might have a similar role in ecosystems as iron, and mechanisms for zinc sequestration have been recently reported to further contribute to nutritional immunity by similar mechanisms as iron sequestration [14–16].

Bacterial extracellular proteins are either actively transported through the cytoplasmic membrane into the environment or simply shed from the surface. The composition of the extracellular proteome, also known as the secretome, strongly depends on the nutrient preferences of the bacterium [17]. Bifidobacterial secretomes have been used to study diversity and physiology of the genus, and to identify differences in nutrient uptake and stress response [18, 19]. Extracellular proteins of bifidobacteria are pivotal for host interactions and -adaptations, for nutrient uptake, adhesion, and stress sensing [20–24]. Further, extracellular proteins of bifidobacteria are potentially directly involved in mechanisms beneficial to the host [25], and the secretome is therefore an ideal target to understand interactions and responses of bifidobacteria in the GI-tract.

Functional genomics is powerful to identify bifidobacterial mechanisms active in the gut, such as genes involved in host-microbe interactions, the degradation of human milk oligosaccharides, or pili encoding genes [10, 23]. Combined genomic and proteomic analyses of bifidobacteria revealed mechanisms of adaptation to the GI-tract and genetic functions that mediate specific host-microbe and microbe-microbe interactions [26, 27]. In contracts to genomics, proteomic detects the functional gene products that are present under specific conditions [28, 29]. Proteomic also allows comparison of strain specific features under similar conditions and is therefore well suited for studying features which may not be accessed by genomics [28]. However, analyses of proteomic mass spectra (MS) rely upon homology with pre-established protein sequences derived from genus related databases [30]. Such approach will inevitably fail to identify strain-unique protein sequences [22] and because many bifidobacterial properties are strain specific, homology-driven proteomics has limits to identify important phenotypic features [19]. A combined genomic-proteomic approach in which the genome is sequenced and used to build a protein database is therefore more suitable for the analyses of the proteomic MS data form bifidobacteria.

*Bifidobacterium kashiwanohense* PV20–2 and *Bifidobacterium pseudolongum* PV8–2 were isolated from the feces of breast-fed, anemic Kenyan infants. These strains exhibit high iron sequestration mechanisms and their whole genomes have been sequenced [12, 31, 32]. In this study, the genomes of *B. kashiwanohense* PV20–2 and *B. pseudolongum* PV8–2 were compared to other bifidobacterial genomes to identify genes potentially involved in iron metabolism. Further, we identified the secretome of both strains under iron limiting conditions using a combined genomic-proteomic approach. In this approach, the predicted coding sequences were used to identify MS/MS-peptides obtained via a 1D gel-based shotgun proteomic approach.

## Results

### Comparative genome analyses of *B. kashiwanohense* PV20–2

*B. kashiwanohense* PV20–2 (PV20–2) and *B. pseudolongum* PV8–2 (PV8–2) were selected for their high iron sequestration in a screening of 56 bifidobacterial strains isolated from the feces of anemic infants in Kenya [12]. To analyze whether specific adaptations related to iron uptake were present in the genomes, we compared the complete genomes of PV20–2 and PV8–2 to 82 completely or partially sequenced bifidobacterial genomes (Additional file 1: Table S1).

The genome of PV20–2 contains 2077 CDSs. A comparison to 82 bifidobacterial genomes, including that of *B. kashiwanohense* strain DSM 21854, revealed 58 CDSs unique for PV20–2, of which 55 encode hypothetical functions (Additional file 2: Table S2). Interestingly, the two unique CDSs AH68_05490 and AH68_05500 are located in a region containing genes that are not or rarely found in other bifidobacteria (region AH68_05460–05520). Refined analyses of the genes in this region revealed that they have high homology to non-ribosomal peptide synthesis genes. Moreover, a conserved domain search [33] shows that the protein encoded by AH68_05485 contains a non-ribosomal peptide synthesis domain (e-score = 9.28e-127). This protein has only one poor hit in the genome comparison, in *Bifidobacterium adolescentis* L2-32 (e-score = 3.14e-14). The 560 AA at the C-terminus of this CDS share 33% identity and 52% similarity with a bacillibactin synthetase from *Bacillus subtilis* 168 [34], and 26% identity and 48% similarity with a pyoverdine synthetase from *Pseudomonas aeruginosa* [35], both enzymes involved in siderophore biosynthesis. Upstream of the putative siderophore synthesis genes an ABC-transporter is located (AH68_05505).

**Fig. 1:**
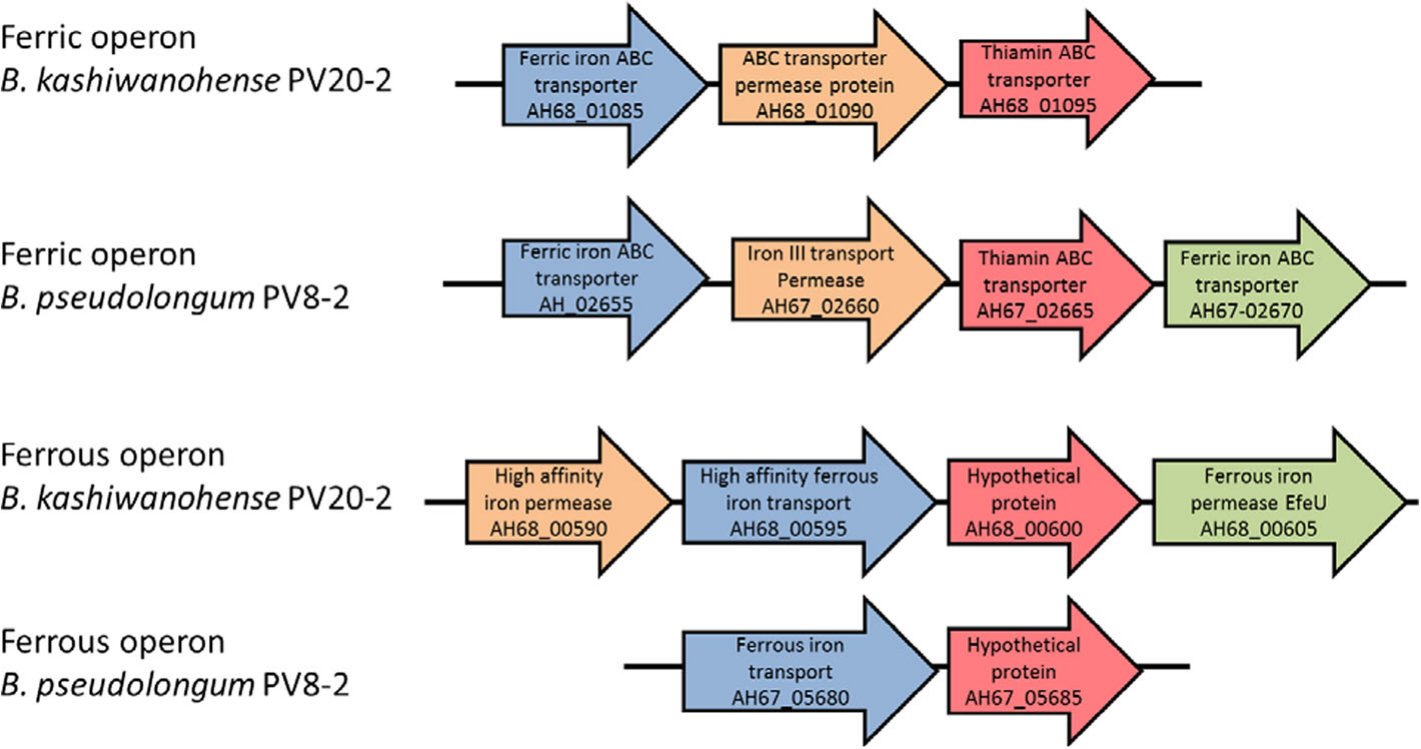
Ferric and ferrous operons identified in the genome of *B. kashiwanohense* PV20-2 (AH_68) and *B. pseudolongum* PV8-2 (AH_67). Homologous genes are indicated by the same colors

Additionally, PV20-2 was compared solely to the type strain *B. kashiwanohense* DSM 21854 [23]. PV20-2 contained 252 proteins not found in DSM 21854. Of these proteins, 197 were found in one or more of the 82 bifidobacterial genomes used in this study (Additional file 1: Table S1 and Additional file 2: S2). Of these 197 CDSs, 47 had highest homology to proteins in *B. longum* strains, followed *by Bifidobacterium adolescentis* (32 CDSs), *Bifidobacterium pseudocatenulatum* (24) and *Bifidobacterium breve* (22). Finally, PV20–2 contained a ferrous and a ferric operon (Fig. 1), both also present in strain DSM 21854.

### Comparative genome analyses of *B. pseudolongum* PV8–2

**Fig. 2:**
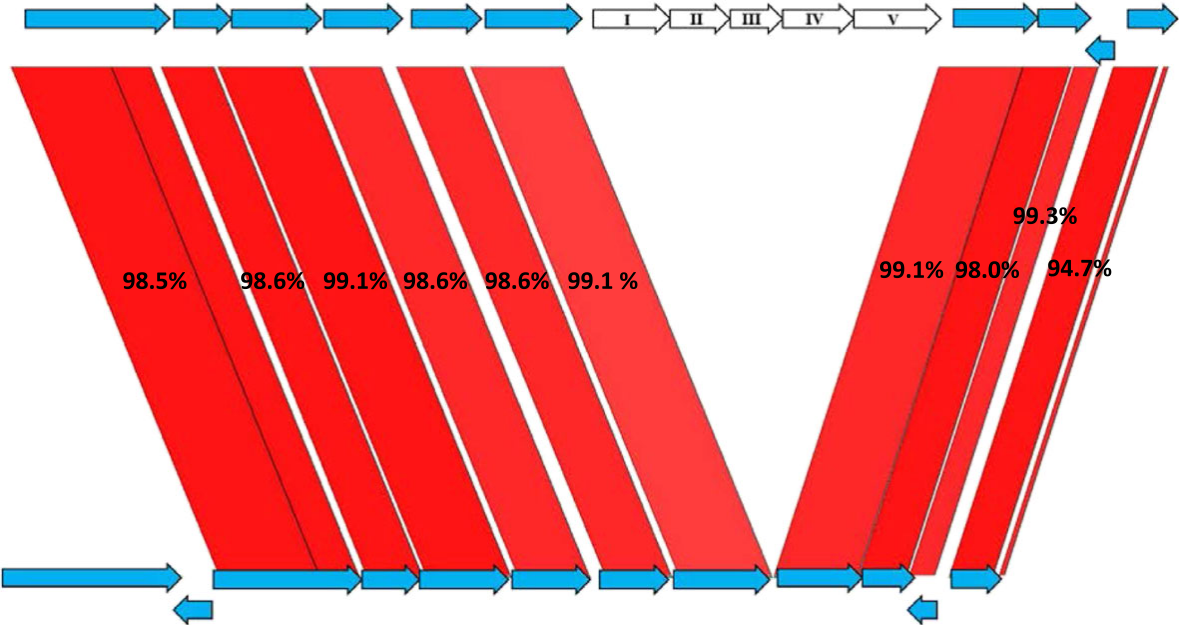
Genetic organization of the ferric operon of *B. pseudolongum* PV8-2 and comparison of the region to *B. pseudolongum* AGR2145. Homologous genes are linked with red lines and their degree of identity provide as percentage of identity. The inserted genes in *B. pseudolongum* PV8-2 are indicated in white: I: Ferric iron ABC transporter, iron-binding, II: IRON(III)-Transport system permease protein, III: Thiamin ABC transporter, transmembrane, IV: Ferric iron ABC transporter, ATP-binding protein, V: COG family: predicted phosphohydrolases

In parallel, *B. pseudolongum* PV8–2 was compared to the 82 bifidobacterial genomes and separately to the two available genomes of the species; *B. pseudolongum* AGR 2145 (fecal calf isolate) and *B. pseudolongum* subsp. *globosum* DSMZ 20092 (rumen isolate). The genome of PV8–2 harbored 1552 protein encoding genes of which 22 were not found in any other bifidobacterial genomes (Additional file 3: Table S3). The products of these 22 CDS encoded hypothetical or phage related functions. A total 78 CDSs did not have homologs in the two *B. pseudolongum* strains, of which 56 were found in other bifidobacterial genomes (Additional file 3: Table S3). Two larger insertions were found in the PV8–2 genome. One insertion encodes genes for arabinogalactan transport and utilization (AH67_01080–01120) that are organized in a similar order as in *B. adolescentis* ATCC 15703 (data not shown). In addition, two galactosidases (AH67_0181 and AH67_1596) were identified in PV-8–2 and not in the other *B. pseudolongum* strains, which might be involved in degradation of the galactose moiety of arabinogalactan. A second insertion contains an iron ABC transporter operon consisting of a ferric iron binding protein (AH67_02660), two membrane components (AH67_02665 and 02670), and the ATP-binding protein (AH67_02675). Detailed comparison of the corresponding genome regions of the three *B. pseudolongum* strains showed a highly conserved organization, with exception of the iron operon (Fig. 2), strongly suggesting that the operon has been acquired by PV8–2. The GC -content of the insertion is 59.4%, only slightly lower than that of the genome (62.4%). The encoded proteins were found only in an unknown faecal isolate belonging to the Actinobacteria phylum and in the rumen isolate *Bifidobacterium* sp. AGR2156 (cut off 80% similarity).

Besides the ferric iron operon described above, also a ferrous iron transporter was predicted in PV8–2 (AH67_05680, Fig. 1). This transporter is also found in the other two *B. pseudolongum* strains and a BLAST search revealed that it is present in many bifidobacteria (data not shown).

### Secretome analysis of *B. kashiwanohense* PV20–2 and *B. pseudolongum* PV8–2 under low iron conditions

**Fig. 3:**
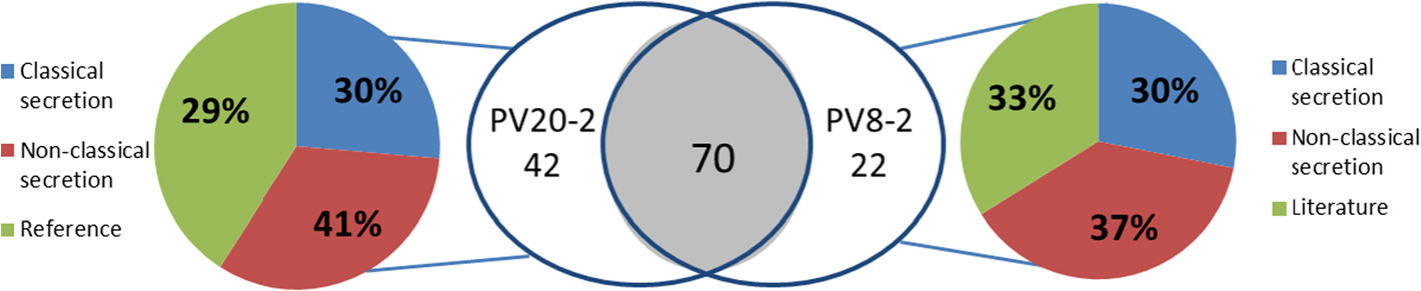
Distribution of proteins in the secretome of PV20-2 (*B. kashiwanohense* PV20-2) and PV8-2 (*B. pseudolongum* PV8-2). Colored circles show the fraction of proteins secreted by classical secretion pathway (blue), non-classical secretion pathway (red) and identified in literature (green). Right circle, represent the percentage of proteins identified in PV8-2 secreted classical secretion pathway (N-terminal sequence, blue), non-classical secretion pathway (N-terminal sequence, red) and the ones identified in literature (green)

The iron uptake mechanisms of PV20–2 and PV8–2 were further studied by secretome analyses during growth under low iron conditions. The strains were grown in a chemically semi-defined medium with a low iron concentration of 1.5 μM [12] and extracellular proteins were isolated and identified by LC/MS. A total of 112 proteins were identified in the secretome of PV20-2 (Additional file 4: Table S4) of which 34 (30%) were predicted to contain a classical signal peptide, 46 (41%) were predicted to be secreted by a non-classical secretion pathways, and 32 (29%) were previously reported to be extracellular (Fig. 3). In PV8–2, 92 proteins were identified in the exoproteome (Additional file 4: Table S5). Of these, 28 (30%) were predicted to contain a classical signal peptide, 34 (36%) were predicted to be secreted by a non-classical secretion pathway and 30 (33%) were previously reported to be extracellular (Fig. 3). Of the 112 identified proteins in PV20–2, 66 proteins had a homologue in the secretome of PV8–2 (Table 1).

**Table 1:** Proteins identified in the exoproteome of *B. kashiwanohense* PV20-2 with an homologue in *B. pseudolongum* PV8-2

Locus tag	Assigned function	Homologue in PV8-2	SP^a^	Secreted^b^
AH68_05990	membrane protein	AH67_04310		x
AH68_00170	serine/threonine protein kinase	AH67_00415		x
AH68_00180	penicillin-binding protein	AH67_00425	x	x
AH68_00315	peptidase	AH67_00490		x
AH68_00635	membrane protein	AH67_00835		x
AH68_00655	peptidase C69	AH67_00865		x
AH68_00900	penicillin-binding protein	AH67_01125		x
AH68_00945	phosphotransferase	AH67_01155		
AH68_01075	glycerol-3-phosphate acyltransferase	AH67_01265		
AH68_01345	glucose-6-phosphate isomerase	AH67_01530		
AH68_01575	phosphate ABC transporter substrate-binding protein	AH67_01870	x	x
AH68_01610	50S ribosomal protein L7	AH67_01905		
AH68_01655	molecular chaperone GroES	AH67_01950		
AH68_01785	50S ribosomal protein L4	AH67_02085		
AH68_01885	adenylate kinase	AH67_02185		
AH68_02020	hypothetical protein	AH67_02295	x	x
AH68_02050	PTS mannose transporter subunit IIABC	AH67_02320		x
AH68_02165	amidase	AH67_02505		x
AH68_02170	peptidase P60	AH67_02510	x	x
AH68_02465	branched-chain amino acid ABC transporter substrate-binding protein	AH67_02445	x	x
AH68_02570	amidase	AH67_03020	x	x
AH68_02580	ABC transporter substrate-binding protein	AH67_03110	x	x
AH68_02785	amino acid ABC transporter substrate-binding protein	AH67_06815	x	x
AH68_02795	methionine ABC transporter ATPase	AH67_06850	x	x
AH68_02900	arylsulfatase	AH67_03300		x
AH68_03085	elongation factor G	AH67_03425		
AH68_03090	elongation factor Tu	AH67_03430		
AH68_03095	elongation factor P	AH67_03450		x
AH68_03260	molecular chaperone GroEL	AH67_03615		
AH68_03390	ABC transporter substrate-binding protein	AH67_03690	x	
AH68_03430	Clp protease	AH67_05875		
AH68_03845	hypothetical protein	AH67_05645		x
AH68_03865	enolase	AH67_05625		
AH68_03915	transcription elongation factor GreA	AH67_05585		
AH68_03955	transcriptional regulator	AH67_05555	x	x
AH68_03970	hypothetical protein	AH67_05545	x	x
AH68_04025	ABC transporter substrate-binding protein	AH67_05490	x	x
AH68_04165	acetate kinase	AH67_05125		
AH68_04240	hypothetical protein	AH67_05000		x
AH68_04280	hypothetical protein	AH67_04940		x
AH68_04600	ABC transporter substrate-binding protein	AH67_00280	x	x
AH68_05530	choloylglycine hydrolase	AH67_04680		x
AH68_05760	transaldolase	AH67_04590		
AH68_05765	transketolase	AH67_04585		
AH68_06400	glutamine synthetase	AH67_03825		
AH68_06950	peptidylprolyl isomerase	AH67_05945		
AH68_07210	ABC transporter substrate-binding protein	AH67_06260	x	x
AH68_07300	glyceraldehyde-3-phosphate dehydrogenase	AH67_06350		
AH68_07370	peptide ABC transporter substrate-binding protein	AH67_06260	x	x
AH68_07705	Lon protease	AH67_06760		x
AH68_08215	hypothetical protein	AH67_07275		x
AH68_08365	glycine cleavage system protein H	AH67_07395		
AH68_08385	thioredoxin	AH67_07415		
AH68_08390	hypothetical protein	AH67_07420		x
AH68_08925	hypothetical protein	AH67_07735	x	x
AH68_08940	peptidylprolyl isomerase	AH67_07745	x	x
AH68_08975	ATP synthase F0F1 subunit B	AH67_07780		x
AH68_09035	inorganic pyrophosphatase	AH67_07840		
AH68_09070	hypothetical protein	AH67_07875		x
AH68_09115	glutamyl-tRNA amidotransferase	AH67_07920		
AH68_09245	single-stranded DNA-binding protein	AH67_08040		x
AH68_09250	30S ribosomal protein S6	AH67_08045		
AH68_09340	phosphoglycerol transferase	AH67_08095		x
AH68_09765	heat shock protein GrpE	AH67_08330		x
AH68_09770	molecular chaperone DnaK	AH67_08335		x
AH68_10135	sugar ABC transporter ATP-binding protein	AH67_08590		

^a ^signal peptide predicted according to [22]

^b^as predicted by SecretomeP 2.0

Remarkably, the secretome of PV8–2 contained a ferric - iron transporter (AH67_02660) but no ferric transporter was detected. In contrast, secretome of PV20–2 contained three ferrous iron transporters AH68_00590, AH68_00595 and AH68_00600. In addition, the ABC-transporter genetically linked to the putative siderophore genes (AH68_05505) was found. Among the proteins identified in both secretomes were 3 elongation factors, a glyceraldehyde-3-phosphate dehydrogenase, proteins related to stress and transporters, including the zinc ABC transporter ZnuA (Table 1). A second protein with zinc binding function (ZinT, locus tag AH67_08575) was identified in PV8–2 secretome (Additional file 4: Table S5). Further, some clear surface related proteins were identified such as a penicillin binding protein and a membrane protein. In the exoproteome of PV8–2 a lysozyme M1 (AH67_08110) was identified.

## Discussion

In this study we analyzed two bifidobacteria strains that had the highest iron sequestration capacity among 56 bifidobacterial isolates from stools of iron - deficient Kenyan infants [12]. Using a comparative genomics and a combined proteomic-genomic approach we identified in both strains specific adaptations to the low iron concentration encountered in the GI-tract of iron-deficient infants.

The comparative genome analyses revealed adaptations to low iron conditions and to a specific diet, thus the genomic content reflects the source of the strains, as already proposed previously for bifidobacteria [36]. *B. kashiwanohense* PV20–2 encodes genes putatively involved in bacillibactin and pyoverdines syntheses that were not present in strain DSM 21854. Bacillibactins and pyoverdines are both siderophores [34, 35] and the corresponding genes in PV20–2 could encode for the synthesis of a bifidobacterial siderophore. The identification in the secretome of an ABC-transporter genetically linked to the putative siderophore genes suggest that this transporter is involved in the uptake of the iron-siderophore complex. In the *B. pseudolongum* PV8–2 strain, iron related genome content included a ferric iron transporter clearly inserted in the genome (Fig. 2). The GC-content of 59.4% of the insertion suggests it has been acquired from a high-GC-content bacterium. It is, however, not known when the insertion occurred and it is therefore also possible that the sequence is already adapted to the host chromosome for its GC-content. The origin of this insertion is therefore not clear. The presence of a siderophore operon in strain PV20–2 and an additional iron transporter in PV8–2 seems directly related to the selective pressure mediated by the low-iron abundance in the gut of the iron-deficient infants.

Arabinogalactan are pectin derived sugars that can be utilized by some bifidobacteria [37] and the corresponding genes have been described in *B. breve* [38]. Arabinogalactan is found in plant cell wall and is a potential prebiotic with some selectivity for bifidobacteria [39] The occurrence of an arabinogalactan machinery in the human isolate PV8–2, and not in the rumen isolates DSM 20092 and AGR 2145 might be related to the specific diet of the Kenyan infant from whom feces the strain was isolated. The infant weaning diet is composed of mother’s milk and maize porridge cooked in water, and therefore containing arabinogalactan-rich source [40].

Approximately 75% of the CDSs that were unique for PV8–2 and PV20–2 in the direct comparison to strains of their own species were found in other bifidobacteria. This suggests that the corresponding genes have been acquired from other bifidobacteria and that horizontal gene transfer (HGT) occurs between members of the phylum. In addition, the ferric iron operon of PV20–2 is inserted in a conserved genome region and likely acquired via HGT. The high cell densities and strong selective pressure in the GI-tract facilitate HGT and outgrowth of novel variants [41, 42]. In addition, costs of gene transfer are lower if the genes fit well in the recipient’s system and transfer occurs therefore easier between related species [43]. The occurrence of a whole set of genes horizontal through the genus strongly suggests that HGT events occur frequently between bifidobacteria. The unique genes of PV20–2 and PV8–2 encoded mostly hypothetical or phage related functions, paralleling an observation in the *B. breve* taxon [44].

Bacteria, including bifidobacteria, secrete proteins to ensure the acquisition of essential nutrients, cell to cell communication, and competition in their respective niches [45]. Extracellular proteins related to iron metabolism, adhesion and antimicrobial activity were identified in the secretome of PV8–2 and PV20–2. Both strains apparently use therefore a combination of strategies to efficiently compete and interact with the environment. This observation is in agreement with recent observations that several mechanisms are used by bacteria to compete in different environments [46–49]. Furthermore, the ability of bacteria to occupy specific environments mainly relies on their ability to obtain adequate supplies of nutrients that are indispensable for their growth, such as iron [50, 51]. In the exoproteome of *B. kashiwanohense* PV20–2 three ferrous iron transporters were identified which seems directly related to the high iron internalization activity of the strain [12]. In contrast, in the exoproteome of PV8–2 a ferric iron transporter was identified. This suggests different iron acquisition strategies in both strains. Remarkably, the ferric transporter of PV8–2 is likely acquired via HGT and its occurrence in the secretome shows that it is active. The absence of ferrous transporter proteins in the secretome suggests that the acquired ferric transporter has taken over the function for iron uptake. The competition for iron within the gut microbiota and the limited availability of iron in the intestine, leads to a constant battle for iron between the enteric microbiota and the host [7, 52, 53]. Thus efficient iron acquisition mechanisms are likely pivotal for proliferation and persistence in the gut [14, 16, 54]. Production of iron binding proteins by bifidobacteria may also limit the iron available for pathogens thus potentially being implicated in a so called nutritional immunity phenomenon [55–57]. In competition, inhibition, and displacement assays using HT29-MTX cell lines, strain PV8–2 reduces adhesion of *Salmonella* Typhimurium and *Escherichia coli* O157:H45 while strain PV20–2 only reduced adhesion of *S*. Typhimurium [58]. This competitive strength of especially PV8–2 might be in part due to iron binding mechanism limiting iron availability for pathogens.

The zinc ABC transporter ZnuA was identified in the exoproteome of both strains. Zinc metal transporters may facilitate, although to a lesser extent, the uptake of other divalent ions, such as iron [59]. Alternatively, their detection may be the result of a common micronutrient stress sensing. Further, ZinT was identified in PV8–2. ZinT forms a complex with ZnuA to enhance its activity. ZinT was identified in the extracellular fraction of enterohemorrhagic *E. coli* where it was important for colonization [60, 61]. Zinc competition in the intestine may also play a role in nutritional immunity and control of enteropathogens growth, but further research is needed to confirm the beneficial effects of zinc sequestration in the gut environment [14, 47].

Elongation factors have been found in the secretome lactobacilli and are involved in the adhesion *Lactobacillus johnsonii* to human intestinal cells and mucins [62, 63]. Their presence in the secretome and the high adhesion properties of both strains [64] suggests a similar role for these factors in bifidobacteria. Similarly, glyceraldehyde-3-phosphate dehydrogenases, and the stress proteins GroEL and DnaK were previously shown to be involved in adhesion of lactobacilli [65–68], and their occurrence in the secretome of PV20–2 and PV8–2 suggests same functionality.

The vast majority of the proteins identified in both secretomes possess an N-terminal signal peptide, and are predicted to be secreted, or were already shown to be extracellular (Additional file 4: Table S4 and S5). In addition, many proteins were predicted to be lipoproteins, while lipoproteins of bifidobacteria were reported to stimulate the immune system and were involved in adhesion [69, 70]. However, the implication of these proteins in more than one function has to be further tested. Furthermore, the lysozyme M1 might be involved in inhibition against competing microorganisms. Lysozymes or glycoside hydrolases such as N-acetylmuramidases are reported to be involved in competition and antimicrobial activity because they can act as bacteriolysins [71].

## Conclusions

In our study we used genomics and proteomics to characterize the response to low iron conditions of *B. kashiwanohense* PV20-2 and *B. pseudolongum* PV8–2, selected for their high iron sequestration properties. The expression of ferric and ferrous binding proteins and the different responses to iron limitation reflect strain specific characteristics, which is in agreement with high strain variation among bifidobacteria. In addition, to the ferrous iron operon identified in the genome of both studied strains, PV8-2 was shown to have a specific ferric iron operon. The iron binding proteins identified in this study are likely involved in iron metabolism. By combining functional assays and genomics and proteomic tools we could uncover iron-sequestration mechanisms that could provide important competition edge for colonization in the low iron environment of the infant gut. However, the specific function of the extracellular proteins disclosed in this study and their role in the GI-tract colonization remain to be investigated.

## Methods

### Strains and growth conditions

*B. kashiwanohense* PV20-2 and *B. pseudolongum* PV8-2 were previously isolated from stool samples of iron deficient Kenyan infants [12]. Pre-cultures were performed in MRS broth (Biolife, Italy), supplemented with 0.5 g/L L-cysteine (Sigma-Aldrich, Switzerland), anaerobically at 37 °C in Hungate tubes with CO_2_ headspace (PanGas, Switzerland). Growth was monitored in a Biowave CO8000 spectrophotometer (Biochrom England). Pre-cultures with an OD_600_ of 1.5 were centrifuged and cells were suspended in peptone water (Oxoid, Switzerland) supplemented with 0.05% L-cystein, pH 6.5. A portion of 3.75 mL of this suspension was used to inoculate 250 mL of a chemically semi-defined medium with an iron concentration of 1.5 μM described previously [12]. The iron concentration in the media was measured using a graphite furnace atomic absorption spectrophotometer (AA-240Z, Varian Inc., Australia) according to manufacturer’s instructions [72]. Cultures were incubated at 37 °C under anaerobic conditions until an OD_600nm_ 1.5 (corresponding to the early stationary phase). Three biological replicates were performed.

### Analyses of the genomes of *B. kashiwanohense* PV20-2 and *B. pseudolongum* PV8-2

The genomes of *B. kashiwanohense* PV20–2 (Genbank accession number CP007456), and *B. pseudolongum* PV8–2 (CP007457) were compared to a local database containing the genomes of 42 completely sequenced and 40 draft genomes of bifidobacteria (Additional file 1: Table S1), using the tBLASTn algorithm [73] embedded in the CLC genomic workbench 7.0 (CLC genomics, Denmark). The E-value cut off was set at < 1exp-4, as was used previously for comparative genomics of bifidobacteria [74]. Comparisons of genetic organization were visualized by the Artemis Comparison viewer [75] with input files that were produced using Double Act version 2 (http://www.hpa-bioinfotools.org.uk/pise/double_act.html).

Coding sequences (CDS) were predicted from the genome and signal peptides were identified using SignalP 4.1 (http://www.cbs.dtu.dk/services/SignalP, [22]) with the option “Gram-Positive” bacteria as organism group, default D-cutoff values (0.45) and transmembrane regions included. Non-classical protein secretion was predicted with SecretomeP 2.0 (http://www.cbs.dtu.dk/services/SecretomeP, [76]) with Gram-positive bacteria selected as organism group. A protein with signal peptide was considered when the SecP score was above 0.5. Lipoproteins were identified using PredLipo with default parameters [77].

### Extraction of extracellular proteins and 1D gel electrophoresis

Cultures were centrifuged at 10,000 × *g* at 4 °C for 10 min and the supernatants were passed through a 0.22 μm pore size filter (Millipore, Switzerland). Proteins in the supernatant were precipitated with 10% v/v trichloroacetic acid (Sigma-Aldrich) at 4 °C for 3 h and harvested by centrifugation at 11,000 × *g* at 4 °C for 30 min. The precipitate was washed twice with ice-cold acetone, dried in a centrifugal vacuum concentrator (Vacufuge 5301, Eppendorf, Switzerland), and stored at −20 °C until further use. Protein pellets were solubilized in 250 μL of 50 mM ammonium bicarbonate at pH 8.5 (AmBic). Protein concentrations were determined according to Bradford using Bradford reagent (Sigma Aldrich) and bovine serum albumin as standard.

Tricine sodium-dodecyl sulfate polyacrylamide gel electrophoresis was used to separate the proteins [78]. The precipitated proteins were mixed 1:1 with Tricine Laemmli sample buffer (Biorad, Switzerland), as described by manufacturer’s instructions, and incubated for 7 min at 99 °C in a Dri-Block (Witec, Switzerland). 50 μg of standardized protein mixture was loaded on a 10–20% Tris-Tricine Mini-Protean TGX precast gel (Biorad), with tris-tricine as running buffer (Biorad, Switzerland). The gel was run at 180 V for 40 min, stained with Coomassie blue G250 and destained with 10% (v/v) acetic acid (Sigma-Aldrich). Gel lanes were cut in eight fractions and prepared for mass spectrometry analysis as described previously [79], with slight modifications. Briefly, gel fractions were washed twice with 50 mM AmBic / 5% acetonitrile (ACN) and dehydrated using 100% ACN. Subsequently, gel pieces were treated with sequencing grade trypsin (Promega, Switzerland) for 1 h at 4 °C according to manufacturer’s instructions. Thereafter, 50 μl digestion buffer containing 50 mM AmBic, pH 8.5 was added, followed by incubation at 37 °C for 16 h. The generated tryptic peptides were extracted with 0.1% trifluoroacetic acid/50% ACN v/v at 37 °C in an ultrasonicator (VWR, Switzerland) for 15 min and finally concentrated with a vacuum concentrator. Peptides were desalted using ZipTip C_18_ micro-columns (Millipore, Switzerland) following the manufacturer’s instructions, and finally dried in a vacuum concentrator. The dried peptide mixture was suspended in 5% ACN/0.1% formic acid v/v for LC-MS/MS measurements.

### Acquisition of proteome data

All data were acquired on an LTQ OrbitrapVelos mass spectrometer connected with an Easy-nLC 1000 HPLC system (Thermo Fisher Scientific, Germany). Peptide samples (4 μL) were loaded onto a frit column (75 μm inner diameter) packed with reverse phase C18 material AQ with 3 μm particle size and 200 Å pore size (Bischoff GmbH, Leonberg, Germany), and eluted with a flow rate of 300 nL per min. Solvent composition of buffer A was 0.1% formic acid in water, and buffer B contained 0.1% formic acid in acetonitrile. The following liquid chromatography gradient was applied: 0 min: 0% buffer B, 90 min: 30% B, 92 min: 100% B, 100 min: 100% B. Survey scans were recorded in the Orbitrap mass analyzer in the range of m/z 300–2000, with a resolution of 30000 at m/z 400. Collision-induced dissociation (CID) spectra were acquired in the ion trap, from the 20 most intense signals above a threshold of 1000 counts. A normalized collision energy of 35% and an activation time of 10 ms were used for CID. The precursor ion isolation width was set to m/z 2.0. Charge state screening was enabled, and single charged ions and unassigned charge states were rejected. Precursor masses already selected for MS/MS acquisition were excluded for further selection during 45 s, and the exclusion window was 20 ppm.

### Protein identification

Peak lists were generated with Mascot Distiller 2.4.1 from Matrix Science using the Thermo MSFile Reader 2.2. Mascot Daemon 2.4 was used for merging and submitting the.mgf files from Xcalibur FT package 2.0.7. The resulting mass spectra files obtained from mass spectrometry analysis were searched using Mascot 2.4.1 (Matrix Science, London, UK) and 2 databases each containing the RAST [80] predicted coding sequences (CDS) of one *Bifidobacterium* strain, i.e., 2,077 CDS for *B. kashiwanohense* PV20–2 and 1704 CDS for *B. pseudolongum* PV8–2. Mascot search parameters were tryptic peptides with a maximum of one missed cleavage during proteolytic digestion, mass tolerances of 7 ppm on the parent ion and 0.7 Da on the MS/MS. Oxidation of methionine (M) and pyroglutamate formation from N-terminal glutamine (N-term Q) were set as variable modifications. Scaffold 4.3 from Proteome Software was used to validate MS/MS based peptide and protein identifications using Mascot and X!Tandem cyclone 2010.12.01.1. Protein probabilities were assigned by the Protein Prophet algorithm [81]. Peptide and protein identifications were accepted when less than 1% False Discovery Rate was achieved. The function MudPit was used to group the eight fractions in each of the three biological replicates. Finally, a protein was considered valid when it was identified in all three biological replicates, at least two different peptides were detected, a signal peptide with one of the two predictors was found and/or reported to be extracellular.

## Additional files

Additional file 1: “PVazquez_etal_additional_file_1”. The file contains the following 2 tables: **Table S1a.** Complete sequenced genomes of bifidobacteria used in this study and their Genbank accession numbers (sheet 1) and **Table S1b.** Contigs of partially sequenced bifidobacteria used in this study and their Genbank accession numbers (sheet 2). (XLSX 62 kb)
Additional file 2: “PVazquez_etal_additional_file_2”. The file contains the following 2 tables: **Table S2a.** Genes uniquely found in PV20–2 when compared to all bifidobacteria (sheet 1) and **Table S2b.** Genes uniquely found in PV20–2 compared to DSM21854 (sheet 2). (XLSX 35 kb)
Additional file 3: “PVazquez_etal_additional_file_3”. The file contains the following 2 tables: **Table S3a.** Genes uniquely found in PV8–2 when compared to all bifidobacteria (sheet 1) and **Table S3b.** Genes uniquely found in PV8–2 compared to AGR 2145 and DSM20092 (sheet 2). (XLSX 13 kb)
Additional file 4: “PVazquez_etal_additional_file_3”. The file contains the following tables: **Table S4.** Proteins identified in the exoproteome of *B. kashiwanohense* PV20–2. **Table S5.** Proteins identified in the exoproteome of *B. pseudolongum* PV8–2. (XLSX 23 kb)

## Abbreviations

GI: Gastro-intestinal
HGT: Horizontal gene transfer
MS: Mass spectra
PV20-2: *B. kashiwanohense* PV20-2
PV8-2: *B. pseudolongum* PV8-2
